# Measuring attention using the Posner cuing paradigm: the role of across and within trial target probabilities

**DOI:** 10.3389/fnhum.2013.00205

**Published:** 2013-05-17

**Authors:** Dana A. Hayward, Jelena Ristic

**Affiliations:** Department of Psychology, McGill UniversityMontreal, QC, Canada

**Keywords:** Posner cuing task, spatial orienting, tonic alertness, voluntary temporal preparation, reflexive attention

## Abstract

Numerous studies conducted within the recent decades have utilized the Posner cuing paradigm for eliciting, measuring, and theoretically characterizing attentional orienting. However, the data from recent studies suggest that the Posner cuing task might not provide an unambiguous measure of attention, as reflexive spatial orienting has been found to interact with extraneous processes engaged by the task's typical structure, i.e., the probability of target presence across trials, which affects tonic alertness, and the probability of target presence within trials, which affects voluntary temporal preparation. To understand the contribution of each of these two processes to the measurement of attentional orienting we assessed their individual and combined effects on reflexive attention elicited by a spatially nonpredictive peripheral cue. Our results revealed that the magnitude of spatial orienting was modulated by joint changes in the global probability of target presence across trials and the local probability of target presence within trials, while the time course of spatial orienting was susceptible to changes in the probability of target presence across trials. These data thus raise important questions about the choice of task parameters within the Posner cuing paradigm and their role in both the measurement and theoretical attributions of the observed attentional effects.

## Introduction

Reflexive orienting acts to interrupt ongoing behavior by rapidly shifting attention toward an unexpected event. In the laboratory, this behavior is typically elicited and measured using the Posner cuing task, where a cue is used to attract participants' attention to a location in space that may contain a response target (e.g., Posner, 1980; Posner and Cohen, 1984). By manipulating the spatial validity between the location of the cue and the location of the target, the Posner cuing task provides a measure of attention by assessing performance for attended targets occurring at the location indicated by the cue (e.g., cued or valid targets) relative to unattended targets occurring at other locations (e.g., uncued or invalid targets). The data generated by this paradigm have contributed immensely to the current understanding of attentional processes, as over the past several decades this procedure has been used to establish a broad base of knowledge across different populations of participants, such as typically developing adults, infants and children, neuropsychological, and psychiatric patients (e.g., Posner et al., 1988; Rafal et al., 1988; Brodeur et al., 1997; Bartolomeo and Chokron, 2002; Bayliss and Tipper, 2006; Ristic and Kingstone, 2009) and various experimental techniques, such as behavioral tasks, EEG, fMRI, and TMS (e.g., Coull and Nobre, 1998; Milliken et al., 2003; Chambers et al., 2004; Channon and Hopfinger, 2011). Furthermore, the data generated by this task have also served as the basis for the development of one of the major theoretical conceptualizations of attentional processes (Posner, 1978; Rafal et al., 1989; Corbetta and Shulman, 2002), establishing the prevailing division between exogenous or reflexive and endogenous or voluntary attentional control (e.g., Jonides, 1981; Berger et al., 2005).

Recent investigations however cast doubt onto the idea that the popular Posner cuing task provides an unambiguous measure of spatial attention (e.g., Tipper and Kingstone, 2005; Gabay and Henik, 2008, 2010). Specifically, extraneous processes that are engaged by the probability of target presence both across and within trials have been found to modulate the spatial attention effects. However, at present it remains unclear whether each of those factors in isolation or in combination influences attentional orienting. To address this question we examined the contribution of across and within trial target probability to reflexive orienting elicited by the Posner cuing task (e.g., Näätänen, 1972; Gabay and Henik, 2008). Understanding the relationship between the parameters of the Posner cuing task and the resultant attentional effects is important for both the theoretical understanding of the measured effects as well as for recognizing the possible limitations of the Posner cuing paradigm as a means of studying spatial orienting in isolation.

### The parameters of the posner cuing task

Since its conception in the 1970's (e.g., Posner, 1978), the Posner cuing task has served as an experimental backbone for eliciting and measuring attention. While over the years researchers have used different variants of this task, a typical sequence of events remains the same: After the presentation of an attentional cue, a target requiring a response is shown at either the cued location or an uncued location after a time delay usually ranging from 100–1000 ms (i.e., cue-target interval). Any performance benefits (e.g., Response Time or RT facilitation) for cued targets are attributed to the cue's ability to engage attention, with the time course of attentional effects revealed by the change in the orienting profile across the cue-target intervals (Klein, 2000; Milliken et al., 2003).

However, in addition to a potential spatial location of the target, the attentional cue in the Posner cuing task also signals the overall probability of target presence *across* all trials as well as *within* each trial. The probability of target occurrence *across* trials is indicated by the rate of overall target presence. Presenting the target on most trials increases participants' readiness to respond to each target event (e.g., Näätänen, 1972) and keeps their level of continuous internal arousal, known as tonic alertness, at a high level (Näätänen, 1970, 1972; Gabay and Henik, 2008; Weinbach and Henik, 2012). When tonic alertness is high, participants' responses are overall fast. In contrast, presenting the target on only a subset of trials decreases participants' overall readiness to respond, lowers their tonic alertness, and leads to overall slower responses (Näätänen, 1972). Thus, the probability of target occurrence *across* trials affects participants' tonic alertness, such that when targets are presented on most trials a state of high tonic alertness leads to increased readiness to respond relative to when targets are presented on a subset of trials, where a state of low tonic alertness leads to diminished readiness to respond.

The probability of target occurrence *within* each trial, on the other hand is affected by the number of targets occurring at each successive cue-target interval relative to the number of total remaining trials (Näätänen, 1970, 1972; Gabay and Henik, 2008). When an equal number of targets are presented at each cue-target interval, which is common practice in the Posner cuing task, the probability that a target will occur within a given trial *increases* as the cue-target interval lengthens. To illustrate, consider an example in which 30 targets are presented at each of four cue-target intervals, and eight trials contain no target, resulting in 128 total trials. Here, the probability of a target appearing at the shortest cue-target interval is *p* = 0.23 (30 out of 128 trials) but as the cue-target interval lengthens and the number of remaining trials decreases (from 128 total trials at the shortest cue-target interval to 38 remaining trials at the longest cue-target interval) the probability of a target appearing at the longest cue-target increases to *p* = 0.79 (30 out of 38 remaining trials; see Figures 2A,C for an illustration). When the sequence of events is presented using this type of distribution in which the probability of *within* trial target occurrence increases with lengthening of cue-target time (often called an “aging” distribution; Näätänen, 1970; Gabay and Henik, 2008), participants form implicit expectations about *when*, within each trial a target is most likely to occur (e.g., Coull et al., 2000). In turn, this temporal expectancy leads to participants' voluntary temporal preparation to respond, which is indexed by the foreperiod effect and overall shortening of RTs with lengthening of cue-target time (Bertelson, 1967). One can equate the probability of target presence within each trial by using a distribution of trials in which the number of targets assigned to each successive cue-target interval reflects one half of the total remaining trials (often called a “non-aging” distribution; Näätänen, 1970; Gabay and Henik, 2008). Using the previous example, with 128 total trials, the probability of target presence within a trial remains set at *p* = 0.5 when the number of targets assigned to each successive cue-target interval is 64, 32, 16, and 8 (i.e., 64/128 total remaining trials at the shortest cue-target interval; 32/64 total remaining trials at the next cue-target interval; 16/32 total remaining trials at the following cue-target interval; and 8/16 total remaining trials at the longest cue-target interval; see Figures 2B,D). Because the likelihood of target presence is equated within a trial, participants form no expectations about when within a trial the target is most likely to occur, their RTs become equated for all cue-target intervals, and the foreperiod effect disappears. Thus, the probability of target occurrence *within* each trial affects participants' voluntary temporal preparation to respond such that increasing the probability of a target's occurrence within a trial induces implicit temporal expectations and faster responses for targets occurring at longer cue-target intervals (i.e., the foreperiod effect).

It is important to note that while the probabilities of target occurrence *across* and *within* trials are integral to the Posner cuing paradigm, the two variables have rarely been manipulated and/or directly measured. Indirect evidence however strongly suggests that the cognitive processes that are engaged by those target probabilities, namely tonic alertness and voluntary temporal preparation interact and depend on similar underlying top-down effects (Callejas et al., 2004; Nobre et al., 2007). This opens up a very real possibility that one or both of these processes aid, influence or interact with the manipulation of spatial validity and the resultant attentional effects, which have typically been attributed to spatial attention alone.

### The influence of the posner cuing task parameters on reflexive attention

Reflexive attention has been extensively studied using the Posner cuing task (Posner et al., 1988; Rafal et al., 1988; Bartolomeo and Chokron, 2002; Milliken et al., 2003; Ristic and Kingstone, 2012). Here, reflexive attention is typically engaged by a peripheral luminance onset, which is spatially uninformative about the upcoming target. Typical results, which have been argued to represent an experimental marker of reflexive attention (Chica et al., 2011) reveal short-lived RT facilitation for cued targets and a lasting inhibition emerging between 300 and 500 ms post-cue (i.e., Inhibition of Return or IOR; Posner and Cohen, 1984; Posner et al., 1985), when target detection responses are measured (Lupiáñez et al., 1997).

Recent studies however indicate that both *across* and *within* trial target probability modulate these typical reflexive effects. Tipper and Kingstone (2005) reported that changing the probability of target presence *across* trials eliminated voluntary temporal preparation and resulted in a decreased magnitude of IOR. IOR magnitude in the baseline condition, in which both the cue and the target appeared on every trial was contrasted with the IOR magnitude in the other four conditions in which either the cue or the target was present in 95% or 75% of trials (e.g., 95% Cue, 75% Cue, 95% Target, 75% Target). A typical foreperiod effect, indexing voluntary temporal preparation emerged in all conditions except when the target was infrequently presented in 75% of trials. Further, it was only in this condition that the magnitude of IOR was attenuated by 11 ms (i.e., by 39%) relative to baseline. Dovetailing with other investigations that have indicated susceptibility of IOR to strategic factors (e.g., Mondor, 1999; Milliken et al., 2003), Tipper and Kingstone concluded that while IOR emerges reflexively, it is modulated by voluntary temporal preparation to respond to the target. In other words, the authors argued that the probability of target presence *across* trials influences both the foreperiod effect and the magnitude of reflexive spatial orienting.

Gabay and Henik (2008) however argued that Tipper and Kingstone's manipulation of reducing the overall number of targets served to lower participants' tonic alertness and not voluntary temporal preparation, as the slowest overall RTs were observed in the critical 75% Target condition (Näätänen, 1970, 1972; Gabay and Henik, 2008). While keeping the probability of target presence *across* trials at a high 94%, Gabay and Henik (2008) manipulated the probability of target presence *within* trials by either using the typical procedure in which the probability of a target within trials increased with lengthening of cue-target interval or by using a distribution in which the probability of a target was equated within trials. They found that while the foreperiod effect was once again eliminated when the probability of target presence was equated within a trial, no change in the magnitude of IOR was observed. This suggests that reflexive spatial orienting is resilient to changes in the *within* trial target probability when the overall probability of target presence *across* trials is high. Put simply, the probability of target occurrence *within* trials did not modulate reflexive orienting, but this effect was only found when the targets occurred frequently overall.

One might notice however that while both Tipper and Kingstone's and Gabay and Henik's manipulations resulted in a decreased magnitude of the foreperiod effect, the experimental manipulations leading to this result were very different. Tipper and Kingstone operationalized voluntary temporal preparation as a decrease in the probability of target presence *across* trials from 95% to 75%. This manipulation resulted in a reduced foreperiod effect and decreased IOR, but those findings might have been due to infrequent overall presentation of the target (i.e., low tonic alertness; Gabay and Henik, 2008). Gabay and Henik (2008, 2010), on the other hand operationalized voluntary temporal preparation as the change in the probability of target presence *within* trials while keeping the overall probability of target presence *across* trials high at 94%. While this manipulation also resulted in a decreased foreperiod effect, in contrast to Tipper and Kingstone, no changes in IOR were observed. However, it is still possible that the resilience of IOR found by Gabay and Henik was due to the overall high probability of target presence across all trials (i.e., high tonic alertness). Thus, it appears that in the Posner cuing task, the probability of target presence *across* and *within* trials influences the foreperiod effect but it remains unclear whether either of these two factors in isolation or in combination affect reflexive spatial orienting.

### The present study

We examined the individual and combined influence of *across* (i.e., tonic alertness) and *within* trial (i.e., voluntary temporal preparation) target probability. We manipulated *across* trials target probability in a same manner as Tipper and Kingstone (2005) by presenting targets (1) frequently on a majority of trials (i.e., 94% Target), leading to high tonic alertness, and (2) infrequently on fewer trials (i.e., 75% Target), leading to low tonic alertness. We manipulated *within* trial target probability in the same manner as Gabay and Henik (2008, 2010) by using a distribution of trials in which the probability of a target within each trial (1) increased with lengthening of cue-target intervals, leading to voluntary temporal preparation, and (2) remained equal across cue-target intervals, leading to diminished voluntary temporal preparation.

If the probability of target presence *across* trials influences reflexive spatial orienting, modulations of reflexive attention are expected only when targets appear overall less often on 75% of trials. If, on the other hand, the probability of target presence *within* trials influences reflexive spatial orienting, modulations of reflexive attention are expected only when targets appear with equal chance at each cue-target interval. If *across* and *within* trial target probabilities influence reflexive orienting in a combined manner, modulations of attentional effects are expected across our conditions. Finally, if reflexive orienting is independent from the Posner cuing task parameters, no changes in the orienting effects are expected.

## Methods

### Participants

Forty-four participants completed the experiment. Twenty-two participants (*N* = 22) were randomly assigned to the high *across* trials target probability group (94% Target) while the remaining twenty-two participants (*N* = 22) were randomly assigned to the low *across* trials target probability group (75% Target).

### Apparatus and stimuli

**Figure 1:**
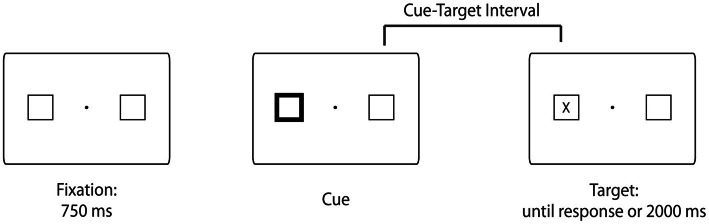
**Example trial sequence.** Trials began with the presentation of a fixation point for 750 ms. Then, one of the placeholders brightened for 80 ms. After the variable cue-target interval (i.e., 106, 372, 638, or 931 ms in the high across trials target probability condition or 106 or 931 ms in the low across trials target probability condition), the target (a capital letter “X”) appeared centered in either the left or right placeholder and remained present on the screen until a response was made or 2000 ms had elapsed. The intertrial interval was 652 ms. Note that the stimuli are not drawn to scale.

Stimuli and an example trial sequence are illustrated in Figure 1. The stimuli were black and white line drawings of a fixation dot, two 2.3° × 2.3° squares presented 5.7° of visual angle to the left and right of fixation, and a capital letter “X” (1°) serving as the target. The stimuli were shown on a 16-in CRT monitor.

### Design

*Across* trials target probability (i.e., tonic alertness) was manipulated between-subjects, while *within* trial target probability (i.e., voluntary temporal preparation), cue-target interval, and cue validity (cued vs. uncued) were manipulated within-subjects. The peripheral cue was spatially nonpredictive, i.e., the target occurred equally often at either of the two spatial locations regardless of the cue's spatial position.

**Figure 2:**
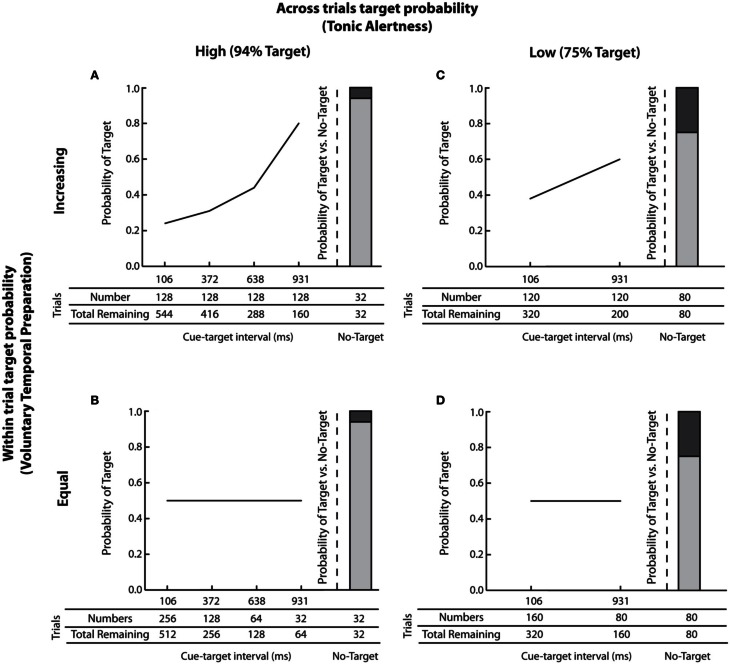
**Illustration of across and within trials target probabilities.** The probability of target presence *across* and *within* trials is presented along with the number of targets assigned to each cue-target interval. **(A)** high *across* trials target probability (i.e., 94% Target) and increasing *within* trial target probability; **(B)** high *across* trials target probability (i.e., 94% Target) and equal *within* trial target probability; **(C)** low *across* trials target probability (i.e., 75% Target) and increasing *within* trial target probability; and **(D)** low *across* trials target probability (i.e., 75% Target) and equal *within* trial target probability.

Figures 2A–D illustrate the probability of target occurrence *across* and *within* trials, and show the number of trials assigned to each cue-target interval. Please note that *across* and *within* trial target probabilities were independent.

#### Across trials target probability

The probability of target occurrence *across* trials was manipulated by changing the number of trials in which a target was presented. High *across* trials target probability, illustrated in Figures 2A,B was implemented by presenting a target in 94% of all trials. This manipulation is known to increase participants' tonic alertness. In contrast, low *across* trials target probability, shown in Figures 2C,D was implemented by presenting a target in 75% of all trials. This manipulation is known to decrease participants' tonic alertness (e.g., Gabay and Henik, 2008).

#### Within trial target probability

The probability of target occurrence *within* trials was manipulated by changing the number of targets presented at each cue-target interval. As depicted in Figures 2A,C, presenting an equal number of targets at each cue-target interval resulted in an increased probability of a target occurring at longer cue-target intervals (c.f., Gabay and Henik, 2008). Figure 2A shows an increasing *within* trial target probability in the high *across* trials probability group. Here 128 targets were assigned to each cue-target interval and 32 trials contained no target, for a total of 544 trials. Thus, the probability of a target *within* a trial increased from *p* = 0.23 at the shortest cue-target interval of 103 ms (128/544 trials) to *p* = 0.8 at the longest cue-target interval of 931 ms (128/160 trials). Corresponding *within* trial target probabilities for the low *across* trials probability group are depicted in Figure 2C.

Equating the *within* trial target probability was implemented differently for the high and low *across* trials target probability groups. This was necessary because the probability of target presence within trials is mathematically determined both by the number of trials that contain a target *and* the number of individual cue-target intervals (Näätänen, 1970). Thus, for the high *across* trials target probability group, when targets occurred frequently in 94% of trials, four cue-target intervals of 106, 372, 638, and 931ms were used, as shown in Figure 2B. Here, the number of targets assigned to each successive cue-target interval reflected half of the total remaining trials, equating the *within* trial target probability to *p* = 0.5. Thus, 256 targets were presented at the shortest cue-target interval of 106 ms (out of 512 total trials); 128 targets were presented at the cue-target interval of 372 ms (out of 256 remaining trials); 64 targets were presented at the cue-target interval of 638 ms (out of 128 remaining trials), and 32 targets were presented at the cue-target interval of 931 ms (out of 64 remaining trials). The target was not shown in 32 trials (i.e., 6% no-target trials or 94% Target trials). Corresponding *within* trial target probability for the low *across* trials probability group when targets occurred infrequently in 75% of trials is shown in Figure 2D. Here, two cue-target intervals of 106 and 931 ms were used, with 160 trials presented at the short cue-target interval of 106 ms (out of 320 total trials) and 80 trials presented at the long cue-target interval of 931 ms (out of 160 remaining trials), thus once again equating the within trial target probability at *p* = 0.5. The target was not shown in 80 trials (i.e., 25% no-target trials or 75% Target trials). Note that, controlling for any potential differences in the temporal profile of orienting, the range of cue-target intervals (i.e., from 106 to 931ms) was constant across all experimental manipulations (Cheal and Chastain, 2002).

Participants assigned to the high *across* trials target probability group completed an average of 528 experimental trials while participants assigned to the low *across* trials target probability group completed an average of 320 experimental trials. Trials were divided over two testing blocks. *Within* trial target probability conditions were blocked and randomized across participants with all other variables manipulated in a pseudorandom order within each participant. An increased number of trials in the high *across* trials target probability group was necessary in order to create the required distribution^1^. Five practice trials were run at the start of the experiment.

### Procedure

The experimental sequence of events was consistent with past studies of reflexive orienting (e.g., Posner and Cohen, 1984; Milliken et al., 2003). Each trial began with the presentation of a central fixation dot, which was flanked by two placeholders for 750 ms. Next one of the two peripheral boxes brightened for 80 ms (accomplished through changing the box line thickness from 1 to 5 points). After the variable cue-target interval, the target appeared centered within either the left or the right placeholder, and participants were instructed to press the spacebar as quickly as possible once they detected the onset of the target (Figure 1). The target remained on the screen until a response was made or until 2000 ms had elapsed. RT was measured from target onset. Participants viewed the stimuli from an approximate distance of 57 cm. They were informed that the cue did not predict the location of the target, were asked to maintain central fixation, and to respond as quickly and as accurately as possible.

## Results

Anticipatory responses (RT < 100 ms), timed out responses (RT > 1000 ms), false alarms, and incorrect key presses were classified as errors. Overall response accuracy was high, exceeding 98% in each experimental condition. Error rates did not vary between groups and across conditions, as verified by a mixed effects ANOVA conducted on interparticipant mean accuracy as a function of *across* trials target probability included as a between-subjects variable, and *within* trial target probability, cue-target interval, and cue validity, included as within-subjects variables. Only a main effect of cue-target interval [*F*_(1, 42)_ = 5.29, *p* < 0.05] was found, indicating more accurate responses at the early relative to the late cue-target interval (all other *Fs* < 4, *ps* > 0.05).

Mean correct RTs were analyzed at the common cue-target intervals of 106 and 931 ms. This approach facilitated direct comparisons of spatial orienting as a function of both *across* trials target probability (i.e., tonic alertness) and *within* trial target probability (i.e., voluntary temporal preparation)^2^. An omnibus mixed effects ANOVA was used with *across* trials target probability (high: 94% Target vs. low: 75% Target) included as a between-subjects variable, and *within* trial target probability (increasing vs. equal), cue-target interval (106 vs. 931 ms), and cue validity (cued vs. uncued) included as within-subjects variables.

**Figure 3:**
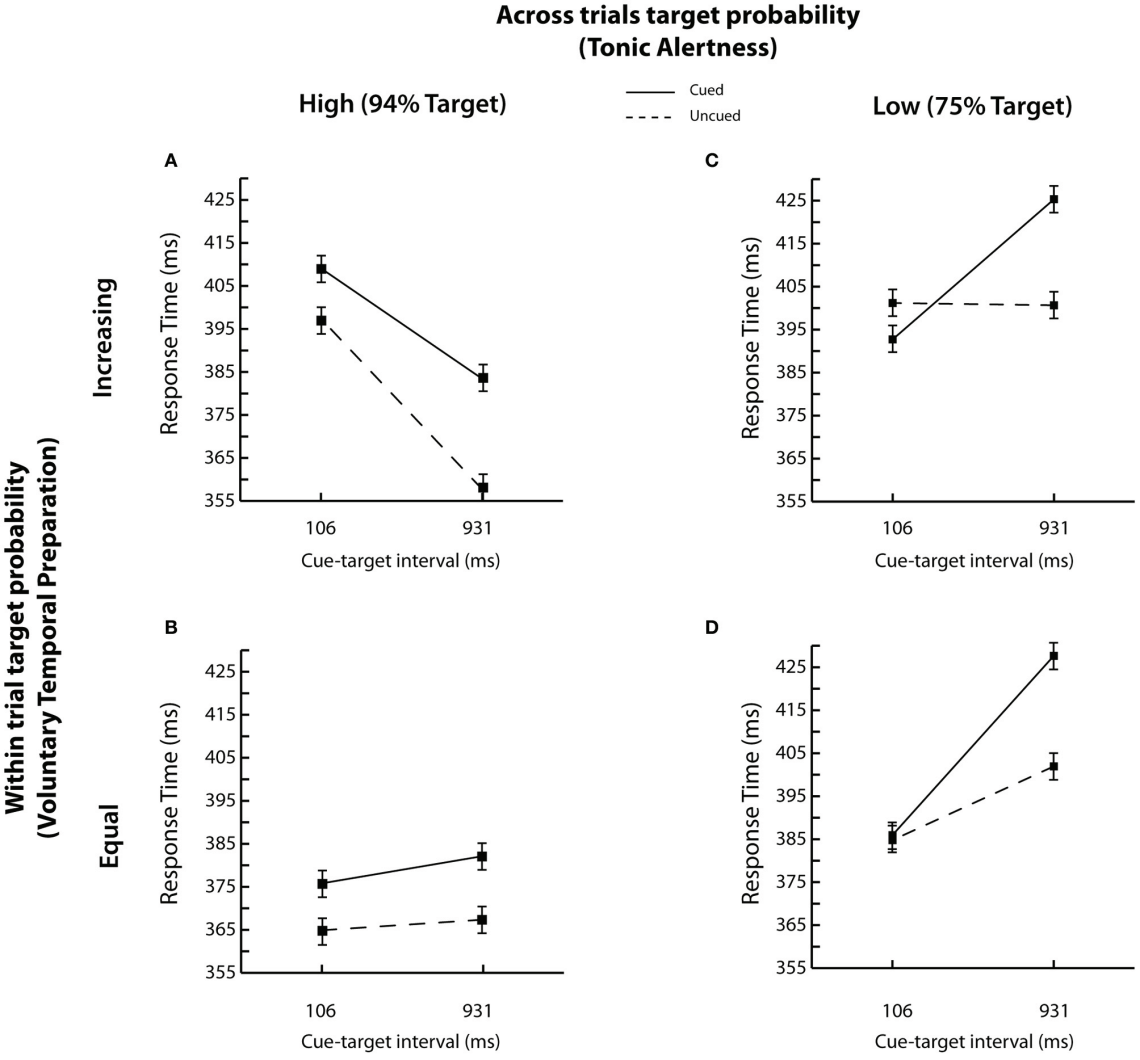
**RT results.** Mean correct RTs as a function of cue validity (cued vs. uncued) and cue-target interval are shown for each of the four experimental conditions: **(A)** high *across* trials target probability (i.e., 94% Target) and increasing *within* trial target probability; **(B)** high *across* trials target probability (i.e., 94% Target) and equal *within* trial target probability; **(C)** low *across* trials target probability (i.e., 75% Target) and increasing *within* trial target probability; and **(D)** low *across* trials target probability (i.e., 75% Target) and equal *within* trial target probability. Error bars represent the standard error of the difference between the means.

Interparticipant mean RTs as a function of *across* trials target probability (i.e., tonic alertness) and *within* trials target probability (i.e., voluntary temporal preparation), cue-target interval, and cue validity are shown in Figures 3A–D.

### The effects of target probabilities on spatial orienting

The key question of interest was whether changes in target probability *across* and *within* trials affected reflexive spatial orienting. Our data revealed that both the time course and the magnitude of spatial orienting were modulated. First, overall we found that the peripheral cue elicited an IOR effect, with faster RTs for uncued relative to cued targets [384 vs. 399 ms; cue validity; *F*_(1, 42)_ = 27.08, *p* < 0.001; Posner and Cohen, 1984]. When the probability of target presence *across* trials was high, inhibition was present at both the short and long cue-target interval, as depicted in Figures 3A,B. However, IOR emerged only at the long cue-target interval when the probability of target presence *across* trials was low, as shown in Figures 3C,D [across trials target probability x cue-target interval × cue validity; *F*_(1, 42)_ = 8.08, *p* < 0.01]. Second, the overall magnitude of spatial orienting was reduced from −18.7 to −8.1 ms for low relative to high *across* trials target probability group when the trial sequence mirrored Tipper and Kingstone's procedure (2005), i.e., when the probability of target presence also increased within trials. In contrast, no reliable changes in the magnitude of reflexive orienting were found (−18.7 to −12.9 ms) when within trial target probability was equated, and the trial sequence mirrored Gabay and Henik's procedure (2008), i.e., when the target also occurred frequently overall [across trials target probability × within trial target probability × and cue validity; *F*_(1, 42)_ = 5.77, *p* < 0.05; all other *Fs* < 2.5, *ps* > 0.1].

Thus, the probability of target presence *across* trials affected spatial orienting as a function of the probability of *within* trial target presence. To probe this finding, we analyzed responses for the high and low *across* trials target probability groups separately as a function of *within* trial target probability, cue-target interval, and cue validity. Reliable inhibition at both the short and long cue-target intervals (100 ms: −8.5 ms; 931 ms: −30.1 ms; both *ts* > 2, *ps* < 0.05, based on paired, two-tailed *t*-tests) with no interactions was found when the targets occurred frequently across trials [*F*_(1, 21)_ = 2.30, *p* = 0.14]. However, a typical time course of spatial orienting with IOR emerging at the late cue-target interval of 931 ms [100 ms: −3.65 ms; *t*_(43)_ = −1.0, *p* > 0.3; 931 ms: −25.2 ms; *t*_(43)_ = 9.12, *p* < 0.001, paired, two-tailed *t*-tests] was found when trials occurred less frequently overall [cue validity × cue-target interval; *F*_(1, 21)_ = 58.73, *p* < 0.001].

Therefore, our findings replicate both Tipper and Kingstone (2005) and Gabay and Henik's (2008) results and additionally reveal that the probability of target presence *across* trials (i.e., tonic alertness) modulates the time course of spatial orienting, but that the joint effect of *across* and *within* trial target probabilities modulates the magnitude of spatial orienting.

### The influence of target probabilities on the foreperiod effect

Next, we examined the influence of target probabilities on the foreperiod effect. While overall the foreperiod effect was not reliable [*F* < 1.5, *p* > 0.2], it was modulated by both *across* and *within* trial target probability. Specifically, and replicating previous research, the magnitude of the foreperiod effect was reduced when *across* trials target probability was low [across trial target probability × cue-target interval, *F*_(1, 42)_ = 20.60, *p* < 0.001] as depicted in Figures 3C,D, and when the probability of target presence *within* trials was equated [within trials target probability × cue-target interval, *F*_(1, 42)_ = 30.43, *p* < 0.001] as shown in Figures 3B,D. Furthermore, a typical foreperiod effect was observed only when both the probability of target presence *across* trials was high and the probability of target presence *within* trials increased with lengthening of cue-target time, as shown in Figure 3A [across trials target probability × within trial target probability × cue-target interval; *F*_(1, 42)_ = 6.71, *p* < 0.05]. These parameters correspond to the task settings typically employed in Posner's cuing procedure (e.g., Buckolz and Rodgers, 1980; Niemi and Näätänen, 1981; Posner and Cohen, 1984). Finally while the omnibus ANOVA returned a marginal main effect of tonic alertness (*p* = 0.1), a direct comparison between overall RTs using an unpaired two-tailed *t*-test revealed significantly slower RTs when the target was overall less likely to appear in the low *across* trials probability group (403 ms) relative to when it was overall more likely to appear in the high *across* trials probability group [380 ms; *t*_(350)_ = −22.8, *p* < 0.001]. Thus, once again replicating the data from both Tipper and Kingstone (2005) and Gabay and Henik (2008) within a single experiment, we found that the magnitude of the foreperiod effect was reduced by decreasing the probability of target occurrence both *across* and *within* trials.

### Magnitudes of spatial orienting and the foreperiod effect

Finally, we assessed whether the magnitudes of spatial orienting (uncued RT-cued RT) and the foreperiod effect (the average RT at 106 ms—the average RT at 931 ms) varied systematically with changes in target probabilities.

**Figure 4:**
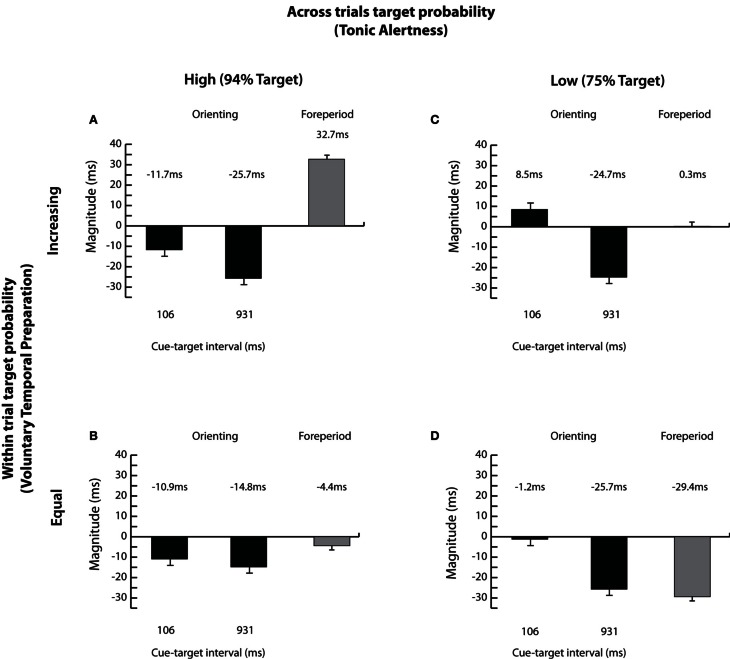
**Magnitudes of orienting and the foreperiod effect.** Magnitudes of orienting (uncued RT—cued RT) and the foreperiod effect (average RT at the 106 ms cue-target interval—average RT at the 931 ms cue-target interval) are shown for each of the four experimental conditions: **(A)** high *across* trials target probability (i.e., 94% Target) and increasing *within* trial target probability; **(B)** high *across* trials target probability (i.e., 94% Target) and equal *within* trial target probability; **(C)** low *across* trials target probability (i.e., 75% Target) and increasing *within* trial target probability; and **(D)** low *across* trials target probability (i.e., 75% Target) and equal *within* trial target probability. Error bars represent the standard error of the difference between the means.

The analysis of orienting magnitudes, illustrated in Figures 4A–D, supported the RT data indicating larger overall inhibition with lengthening of cue-target time [−3.8 vs. −22.7 ms; *F*_(1, 42)_ = 29.16, *p* < 0.001, see Figures 4A–D] with no difference between the magnitudes of orienting with changes in *within* trial target probability (−13.4 vs. −13.2 ms; *F* < 1; Figures 4A,C vs. Figures 4B,D). The early inhibitory effect was present only when the targets occurred frequently overall, i.e., under high tonic alertness, as shown in Figures 4A,B [across trials target probability × cue-target interval; *F*_(1, 42)_ = 8.08, *p* < 0.01], indicating once again that the overall high probability of target presence *across* trials influences the time course of reflexive orienting. Finally, increased overall magnitude of inhibition depended on an interaction between processes engaged by *across* and *within* trial target probabilities emerging only when tonic alertness was high *and* voluntary temporal preparation was induced by the task sequence [across trials target probability × within trial target probability; *F*_(1, 42)_ = 5.77, *p* < 0.05; see Figure 4A].

The magnitude of the foreperiod effect was reduced when targets appeared less often overall [14.5 vs. −14.6 ms; across trials target probability; *F*_(1, 42)_ = 15.56, *p* < 0.001; Figures 4A,B vs. Figures 4C,D] and when the probability of target occurrence was equated *within* trials [16.5 vs. −16.9 ms; within trial target probability; *F*_(1, 42)_ = 58.53, *p* < 0.001; Figures 4A,C vs. Figures 4B,D]. The most pronounced decline in the magnitude of the foreperiod effect was found when both target probabilities were reduced, as shown in Figure 4D. This strongly suggests that both the probability of target presence *across* trials, affecting tonic alertness, and the probability of target presence *within* trials, affecting voluntary temporal preparation, modulate the foreperiod effect.

Taken together, our results indicate that a typical sequence of events in the Posner cuing task, in which the targets appear frequently overall and the probability of target occurrence within a trial increases with lengthening of cue-target time, leads to interactions between tonic alertness and voluntary temporal preparation. This in turn critically modulates both the time course and the magnitude of the measured attentional effects as well as the magnitude of the foreperiod effect.

## Discussion

Motivated by the disparate results showing modulations of reflexive spatial orienting by the processes engaged by *across* and *within* trial target probabilities (Tipper and Kingstone, 2005; Gabay and Henik, 2008), we investigated whether systematic changes in those two parameters affected the resultant measure of reflexive attention. We manipulated *across* trials target probability and *within* trial target probability in isolation and in conjunction, and measured reflexive spatial orienting elicited by a spatially nonpredictive peripheral onset. Our investigation revealed three key findings, which collectively indicate that tonic alertness and voluntary temporal preparation induced by the parameters of Posner's cuing task critically influence the observed attentional effects, which have typically been attributed to spatial orienting alone.

First, we found that an interaction between processes engaged by *across* and *within* trial target probabilities modulates spatial orienting. Overall we found that inhibition was reliable across all experimental conditions. However, the magnitude of inhibition was significantly reduced (i.e., by 10.6 ms) when targets appeared less frequently overall under low tonic alertness. However, this finding held only when at the same time the probability of target occurrence increased within trials. This replicates Tipper and Kingstone (2005), who reported an equal reduction in IOR magnitude (from approximately 30 to 19 ms, cf. Figure 2, Tipper and Kingstone, 2005) using the same manipulation. We also found that overall IOR magnitude was unaffected by the changes in *within* trial target probability. However, this finding held only when the targets appeared frequently overall, replicating Gabay and Henik's (2008) findings. Extending both of those studies we also found that when the probabilities of target presence *across* and *within* trials were reduced in conjunction, the magnitude of spatial orienting was also reduced. Therefore, while in general inhibition appears to be resilient to changes in target probability *across* and *within* trials, its magnitude depends on interactions between the processes engaged by those probabilities, i.e., tonic alertness and voluntary temporal preparation.

Second, changes in *across* trials target probability, or tonic alertness modulated the time course of spatial orienting. Specifically, when targets appeared infrequently in 75% of trials in the low alertness condition, we observed a typical time course of orienting with late emerging IOR. However, an unusual pattern of early and prolonged inhibition was found when targets appeared frequently overall in 94% of trials. This suggests that the inhibitory process might be aided by high tonic alertness, emerging earlier and in a larger magnitude relative to when tonic alertness is reduced. Thus, maintaining high tonic alertness within the Posner cuing task by presenting the response target on the majority of trials appears to fundamentally influence how participants interpret the attentional cue, insofar as the removal of an overall expectancy of a target's presence changes the observed time course of spatial orienting. Conversely, our result showing a typical time course of IOR when targets appeared infrequently in the low alertness case suggests that participants in this condition have either utilized their executive processes to a larger extent because the task required an increased use of response inhibition due to the infrequent presentation of the target, or that they have deployed strategic expectancies in developing inhibitory responses at the long cue-target intervals despite the target's intermittent occurrence. The development of these cognitive expectancies may in turn act to suppress reflexive behaviors like early facilitation, which dovetails with recent results indicating earlier emergence of IOR with increases in tonic alertness (Gabay et al., 2011) and decreases of task difficulty (Van Der Lubbe et al., 2005). It is important to note that unlike IOR, early facilitation is a fragile phenomenon, and has been found to depend on the physical properties of the display (Samuel and Kat, 2003). Thus, the absence of early facilitation in our experiment and perhaps in other studies may reflect its suppression by strategic processes involved in tracking *across* and *within* trial target probabilities. Finally, it is also possible that early facilitation and IOR constitute opposing independent processes (e.g., Tassinari et al., 1994; Danziger and Kingstone, 1998) whereby IOR is mediated by strategic factors (e.g., Milliken et al., 2003) and early facilitation is driven by the cue's physical properties (Pratt et al., 2001; Samuel and Kat, 2003), which might come to the fore when strategic processes are reduced, as it is the case when across trials target probability, i.e., tonic alertness is low. All of these possibilities however suggest a tight coupling between tonic alertness and reflexive spatial orienting, and as such the contributions of each factor within the Posner cuing task should be explicitly examined and dissociated in future studies. In other words, the time course of reflexive attention as measured by the Posner cuing task is directly influenced by the choice of *across* trials target probability irrespectively of the cue's spatial validity.

Third, we found that the magnitude of the foreperiod effect was modulated by both *across* and *within* trial target probability. The foreperiod effect is generally thought to reflect a decreased uncertainty in responding when an advanced warning signal is given to participants (Bertelson, 1967; Niemi and Näätänen, 1981). However, we also found that an overall decrease in target probability *across* trials affected the foreperiod effect. This finding is perhaps due to the fact that in the Posner cuing task the same cue event serves as an indicator of both *across* and *within* trial target probability (Weinbach and Henik, 2012). That is, it appears that the controlled processes, which are driven by those target probabilities, i.e., tonic alertness and voluntary temporal preparation also mediate the foreperiod effect (Callejas et al., 2005). This is supported by our result indicating a typical foreperiod effect only when targets appeared overall more frequently leading to high tonic alertness *and* when the target had an increased probability of occurring with lengthening of cue-target time, while the most pronounced reduction in its magnitude was observed when both of these variables were experimentally reduced.

Thus, the choice of task parameters within the Posner cuing paradigm, specifically those that determine *across* and *within* trial target probabilities, fundamentally influences the observed attentional effects. That is, we were able to modulate both the magnitude and the time course of spatial attention by changing the probabilities of target occurrence while keeping the spatial validity of the cue constant. In particular, we found that the magnitude of spatial orienting was affected by an interaction between tonic alertness engaged by *across* trials target probability and voluntary temporal preparation engaged by *within* trial target probability while the time course of orienting was susceptible to changes in tonic alertness only. Furthermore, we found that the typical foreperiod effect also depended on both tonic alertness and voluntary temporal preparation, suggesting that these two processes share underlying cognitive and executive processes (e.g., Callejas et al., 2004) rather than constituting independent effects (e.g., Fan et al., 2002). These two key results pose interpretational issues for understanding the attentional effects measured by the Posner cuing task and question the attributions of those effects to reflexive or voluntary attentional control (e.g., Posner and Cohen, 1984; Müller and Rabbitt, 1989). That is, it still remains unclear to what extent the magnitude and the time course of reflexive spatial orienting elicited by the Posner cuing task can be attributed to attentional orienting versus processes engaged by the task parameters. One possible reason for the mutual process interference is the fact that in the Posner's cuing task an attentional cue serves multiple purposes—acting as a spatial cue, an alerting signal, and a temporal warning signal. An important aim for future investigations is to dissociate the effects of spatial orienting, tonic alertness, and voluntary temporal preparation within the Posner cuing task, rather than using a set of different tasks (e.g., Fan et al., 2002) by employing distinct events to convey each type of information (see Lawrence and Klein, 2012). The outstanding question is whether and how dissociating the individual contributions of each of these processes will affect the measurement of spatial attention and the resultant theoretical conceptualizations of the attentional effects and their associated control demands (i.e., reflexive and voluntary processes).

### Conflict of interest statement

The authors declare that the research was conducted in the absence of any commercial or financial relationships that could be construed as a potential conflict of interest.

## Footnotes

^1^The magnitude of spatial orienting has been found to vary with increased trial sampling (e.g., Weaver et al., 1998; Pratt and McAuliffe, 1999; Lupiáñez et al., 2001). To examine whether this was the case in our data, we analyzed RTs as a function of the testing block (1^st^ vs. 2^nd^), with *across* trials target probability included as a between-subjects variable, and *within* trial target probability, cue-target interval, and cue validity included as within-subjects variables. Two effects involving testing block were reliable. First, we found a larger reduction in the foreperiod effect in the second block [testing block × cue-target interval; *F*(1, 42) = 6.1, *p* < 0.05]. Second, we found an increased magnitude of IOR in the second block, but only when targets occurred infrequently in 75% of trials [testing block × *across* trials target probability × and cue validity; *F*(1, 42) = 8, *p* < 0.01]. Importantly, a similar finding was not observed when the targets occurred frequently in 94% of trials, which is the condition with more experimental trials (all other effects involving testing block *Fs* < 1.4, *ps* > 0.2). Thus, any differences in spatial orienting as a function of *across* trials target probability are not due to the increased number of trials in the high across trials probability case but likely reflect the development of executive strategies over time when targets occur infrequently.

^2^The same data pattern held when all cue-target intervals were included in the analyses conducted separately for high vs. low *across* trials target probability groups. When targets occurred frequently in 94% of trials, a foreperiod effect was present [*F* (3, 63) = 14.87, *p* < 0.001], participants detected uncued targets faster than cued targets [*F*(1, 21) = 30.44, *p* < 0.001], and responded overall faster when the probability of a target increased *within* trials [*F*(1, 21) = 4.67, *p* < 0.05]. The magnitude of the foreperiod effect decreased when target probability was equated within trials [within trial target probability × cue-target interval; *F*(3, 63) = 18.78, *p* < 0.001] while the IOR effect emerged early and grew in magnitude with cue-target time [cue validity × cue-target interval; *F*(3, 63) = 6, *p* < 0.01; All other *Fs* < 1.8, *ps* > 0.15]. When targets occurred infrequently in 75% of trials, the foreperiod effect was reduced [*F*(1, 21) = 16.50, *p* < 0.001], while spatial orienting [*F*(1, 21) = 13.31, *p* < 0.01] did not vary as a function of *within* trial target probability (*p* > 0.2). Once again, equating the probability of target presence within trials decreased the magnitude of the foreperiod effect [within trial target probability × cue-target interval; *F*(1, 21) = 6.13, *p* < 0.05] while the IOR effect emerged at the late cue-target interval only [cue validity × cue-target interval; *F*(1, 21) = 58.73, *p* < 0.001; All other *Fs* < 3, *ps* > 0.1].
